# Spiritual Pathology; or, the Autobiography of the Insane
†1. Autobiography of the Rev. William Walford. Edited (with a continuation) by the Rev. John Stoughton (of Kensington.) London: Jackson and Walford, St. Paul's Churchyard.—2. Memoir of Richard Williams, surgeon: catechist to the Patagonian Missionary Society in Tierra del Fuego. By James Hamilton, D.D. London: J. Nisbet, 1854.


**Published:** 1854-07-01

**Authors:** 


### ART. IV.—SPIRITUAL PATHOLOGY; OR, THE AUTOBIOGRAPHY OF THE INSANE.*

WE published, in one of our earlier numbers, a short article on the "Autobiography of the Insane," based upon some letters that appeared in the "American Journal of Insanity," written by persons after having recovered from attacks of insanity. Our attention is again directed to this deeply interesting subject by the perusal of the two works whose titles are given at the bottom of this page. It is not our intention at present to enter at any length into a psychological investigation of the facts recorded by those who have attempted to describe their personal feelings and operations of the mind during paroxysms of mental derangement. The subject is too subtle and too profound to be cursorily discussed. Data of this kind cannot be otherwise than invaluable in the hands of those competent, by psychological study and

† 1. Autobiography of the Rev. William Walford. Edited (with a continuation) by the Rev. John Stoughton (of Kensington.) London: Jackson and Walford, St. Paul's Churchyard.—2. Memoir of Richard Williams, surgeon: catechist to the Patagonian Missionary Society in Tierra del Fuego. By James Hamilton, D.D. London: J. Nisbet, 1854.

practical knowledge, to appreciate the phenomena of healthy and morbid mind. It is our intention, therefore, to lay before our readers the salient points contained in the volumes before us, reserving for some other occasion any practical comments that may occur to us in connexion with this important subject.

We do not deem it necessary to detail minutely the facts relating to the early life of Mr. Walford, as recorded in the interesting series of letters published in the volume now under review. We are anxious to confine our attention principally to the psychological portions of Mr. Walford's life, and perhaps, therefore, we may be excused for quoting somewhat in detail his account of his early school-days. We cite the passage with the view of pointing out the grave responsibility incurred by those who undertake the important educational care of the young. The origin of much incurable mental disease may be clearly traced to the badly organised school, and to the criminal and cruel negligence of those whose solemn duty it is to guard the tender minds of the youth placed under their care from vicious habits and moral pollution:—

"The frequent punishments I witnessed in this school, administered often with symptoms of passion amounting almost to fury, terrified me exceedingly at first, but my feelings gradually became less sensitive, till I at length imagined no other means were sufficiently stringent to enforce obedience, and stimulate industry, so that I acquiesced in the propriety of it. This sentiment was very injurious to me, as it greatly strengthened my natural propensity to impatience, and made me too readily susceptible of provocation from imbecility and indolence, an evil to which through life I have been more liable than my conscience and moral sensibility approve. This, however, was far from being the greatest injury I suffered from going to this school. Before that time, I was in a considerable degree ignorant of vice, and unpolluted by its worst seductions, an exemption for which I was indebted to maternal care and guardianship, by the protection afforded to my innocence. As soon as I entered this school, I was very much removed from my mother's inspection, and at no long interval entirely so. Here I came into intimate association with a multitude of boys of all ages, from seven to sixteen.

"Utterly unconscious of the perils to which I was exposed, I easily yielded to the temptations that beset me; and my temper, too susceptible of evil to preserve me from the contagion which surrounded me, quickly rendered me a victim to the abominations that were incessantly before me.

"When reflecting on this part of my history, I cannot avoid deeply feeling the injuries that were inflicted upon me: injuries likely to have spread their pernicious consequences over my whole life, and to have issued in the most fearful results in the life to come.

"I ought undoubtedly, young as I was, to have obeyed the checks of conscience which I occasionally experienced, and to have resisted the inducements to evil which so fatally beset me: but I cannot avoid censuring the neglect of moral discipline in a case where it was so much needed, and where, though it might not have accomplished all that was desirable, would, without doubt, have proved exceedingly beneficial. The master of the school was a clergyman, consequently a teacher of religion and morals; but he was little attentive to the discharge of the obligations of this class to which he had voluntarily subjected himself. It was impossible he could be ignorant of the enormities that were perpetrated within reach of his observation, but which he certainly exerted little or no effort to control."

We now proceed to extract from Mr. Walford's letter those portions that refer directly to his own description of his attack of severe mental disease. In Letter XVI., after referring to his resignation of his pastoral charge at Yarmouth, he observes:—

"I have, hitherto, said nothing respecting an insidious malady, by which, from a very early age, I was often very grievously affected, but of the nature and causes of which I was altogether ignorant, though its effects were inexpressibly painful. This malady had shown itself, chiefly, by almost incessant headaches from my infancy, but soon after my settlement in Yarmouth it assumed a new form. I was attacked by paroxysms of despondency, which during their continuance rendered life a burden almost intolerable. I could give no account of the reasons of such disquietude, and was at a loss to devise any probable means of relief. As, however, it was indispensable to try something, I took a journey on horseback for three or four weeks, and rode three or four hundred miles. The daily exercise, and change of scene and object, greatly relieved me, and at my return I had acquired my usual state of spirits and vigour. But after the interval of a few months, gloom and disquietude again overwhelmed me, and I was constrained to try some amusement that might alleviate the distress, and chase away the clouds. Alternate paroxysms and remissions of this description were experienced during the whole of my abode in Yarmouth. With almost every source of happiness open to me, I was often, for months together, more wretched than I can describe. My prospects were darkened by the thickest clouds, all things present and future were encompassed with fear and dread. Taciturnity, irritability of temper, an unnatural and diseased sensibility of conscience, and such a degree of indolent lassitude as rendered every mental occupation distasteful, increased over me, to such a degree, as to alarm me lest the sanity of my mind should be subverted. At times my thoughts were so agitated and my conceptions so disturbed, as to make me apprehensive that some foreign invisible agency was acting upon me; imaginations of the most extraordinary nature often darted upon me with such rapidity, as left me without control over them.

"I went into company as much as possible, read amusing books, rode much on horseback, but all was in vain; nothing availed to interrupt or divert my thoughts from the most distressing and perplexing difficulties of speculation, as long as the paroxysms continued to exert their power over me. Often I wandered about the fields and country, driven from my occupations and my home, by unutterable anguish, lingering in unfrequented lanes, and hanging on gates and stiles, pouring out frantic and broken supplications to God to have mercy on me. Not seldom I was alarmed lest, in spite of myself, I should abandon all religion, and become an infidel or atheist. I dared not disclose to any the condition of my feelings, lest I should be taken for such, or for a madman. My pious, cheerful, and affectionate wife, was but too sensible that some sad cause of disquietude preyed upon me; but for several years, I replied to her anxious inquiries merely, that my spirits were low and depressed, from what cause I knew not. If these torturing paroxysms had not been relieved by frequent intervals, I must necessarily have relinquished my profession, as it was with inexpressible difficulty I performed its duties, while they were forcibly pressing upon me. So extraordinary, however, was my state, that during the intermissions I experienced, I was often cheerful and even gay; I lost sight of my sorrows, and was astonished at myself that I could ever be so painfully affected. This alternation of feeling, altogether unaccountable to me, continued to actuate me through the whole period of my residence in Yarmouth."

Mr. Walford became one of the classical teachers at Homerton College, and of his residence here he writes :—

"During the first years of my abode at Homerton, I enjoyed many remissions, that were greatly aided by our long vacations, and the journeyings for which they afforded opportunity. Though I had no regular and stated obligation of preaching, I yet was employed on the greater proportion of the Sundays, in delivering one, two, and even three discourses, to congregations in London or the adjacent populous villages. These engagements I found were seldom unaccompanied by advantage to myself, as they interrupted the morbid tendency to gloomy thought and painful speculation, which I had no power of otherwise overcoming. They had frequently a still more beneficial effect, in exciting religious affections, under the influence of which I was induced to hope, with a lively expectation, that I should at length be freed, in the possession of immortal life, from all the sorrows and burdens that now oppressed me.

"Such intervals of delight were very transient, and the next day, often the next hour, found me again plunged into the gloom which had become habitual to me. I had to encounter more than the many evils by which, as I have told, I was oppressed when I lived in Yarmouth; and I repeated the same and other expedients that I at that time adopted, with a forlorn hope, that they might work some relief. The great speculative difficulty respecting the origin of the evil by which, as has been intimated, I had been at various times exceedingly distressed and agitated, returned with such a degree of force, that no means I could employ were able to free me from its perpetual intrusion: at home and abroad, in company and in solitude, it haunted and harassed me, left me no power, with any permanency, to direct my thoughts to other topics, but constrained me to dwell upon it, with scarcely any intermission, at the time when I felt that all my endeavours to solve the mystery were utterly unavailing. No captive loaded with fetters and shut up in the gloom of a dungeon, can more passionately seek for relief than I did, to extricate myself from a bondage which was intolerable. To the anguish occasioned by the incessant occupation of my mind on this one subject, was added a tormenting suspicion, that the Governor of the universe was malevolent, or he would not permit such frightful evils to exist, which he had power at once to terminate. Hence I was involved in never-ending inquiry for some absolute and irrefragable argument in support of the Divine benevolence, as no conception could be fraught with consequences so appalling, as that of irresistible power directed by a disposition to delight in inflicting misery.

"To discover such an argument, I turned over theological and metaphysical volumes of English and Latin writers, more than I am able to enumerate; but the search was vain. If at any time I thought I had grasped a satisfactory theory, my belief in it was evanescent, and it left me helpless as before. I wanted a demonstrative argument; probabilities and moral reasonings appeared to me to be altogether impotent in a case that seemed so flagrant. I exerted my utmost skill to construct a demonstration for myself, but I was unable to succeed. In such a turmoil, the only book that afforded even a temporary relief was Butler's 'Analogy,' to which I continually had recourse whenever I was most heavily oppressed; but the alleviation thus gained speedily forsook me. Besides the incessant agony which was thus inflicted, a morbid restlessness of conscience, which never permitted me to think I made the exertion I might do to promote the welfare, spiritual and temporal, of my fellow-creatures, filled me with most distressing apprehensions respecting the reality of my personal piety, and alarmed me lest I should become subject to the anger of God. Amidst such agitations, tossed as I was from wave to wave of inexpressible distress, I often felt no words could so well describe the horrors of my state as Cowper's lines:—

'Me howling winds drive devious, tempest toss'd,
Sails ript, seams opening wide, and compass lost;
And, day by day, some current's thwarting force
Sets me more distant from a prosperous course.'

To gain some remission of my anguish, I was compelled, when walking or riding alone, to recite mentally verses, English, Latin, or Greek, which I had committed to memory for this purpose,—an expedient not much less annoying than the cogitations that I wished to shun. During many years, I could seldom or never sleep on going to my bed, without adopting this course.

"At length, by the earnest persuasion of a beloved friend, who was, in some degree, acquainted with my disquietudes, I resolved to try what medical aid could do for me; and I applied to a very intelligent and experienced physician for advice, though I augured little advantage from it, as I had a rooted belief that not my body but my mind was in want of healing,—a want not to be redressed by medicine.

"On relating my case as one of extreme dejection, without assigning such particulars as I have detailed, I received a most positive assurance that the malady was derived from the body, and that there was little doubt it would be overcome by suitable curative means.

"No hesitation could exist as to the disinterestedness of the advice, as, on learning from me who I was, the giver of it peremptorily refused any gratuity, and assured me that he should have great pleasure in seeing me, and giving his advice as frequently as I wished. I saw him subsequently many times, always found the greatest kindness and sympathy; but all was, alas! unavailing; as I sunk habitually deeper and more deeply in the slough that on every side environed me. Nothing was now before me but the prospect of being constrained to relinquish my connexion with the College, to abandon all my engagements, and, in obscurity and misery, to await the approach of dissolution, respecting which I entertained the most direful presages.

"In such circumstances, I persisted in pursuing my various occupations, until near the close of my sixteenth year's residence in the College, when, by an unlooked-for and most grievous occurrence, the cup of bitterness, already filled, was made to overflow. My only daughter, of whom I have before made mention as a very engaging, pious, and accomplished child, now about seventeen, met with an accident, which inflicted a wound on the skull, under the effects of which she languished three or four months, when she expired from pressure on the brain, which baffled the exertions of several eminent medical practitioners to relieve. This blow stunned me, in the first surprise occasioned by it: as soon, however, as I could reflect upon it with any degree of calmness, I felt that, deep as was the anguish I suffered from it, it was small compared with that which I experienced from my troubled apprehensions.

"My child was departed from me; yet so contradictory were my feelings, that though my bosom was wrung by alternate paroxysms of doubt akin to atheism, and of imaginations that presented the Governor of the world to me as the adversary, rather than the benefactor and friend of his creatures, I was so awed by the sense of his majesty and wisdom, that, if the lifting up of a finger might have restored to me my much-loved child from the grave, I should have restrained it.

"The influence of the two kinds of distress by which I was affected, differed as much as the causes of it did. My own peculiar suffering never softened my heart, never drew a tear from my eyes,—I was unable to weep, though I often passionately desired to do so: the grief I felt during the time my child was daily sinking to death, and immediately following, vented itself in floods of tears, that seemed to exhaust my whole nature, and render me incapable of repressing them. As soon, however, as 'my dead' was committed to the grave, I resolved instantly to return to the vigorous discharge of my college and other duties, as the surest means of overcoming my sorrows. I went into the lecture-room; but, after one or two attempts, I found resistance vain; and, to change the scene, went into the country to visit a friend, by whose converse I had often been cheered, and of whose sympathy I was fully assured. I should now terminate my narrative if I were not actuated by a hope that a perusal of what is to follow may afford some support and relief to any of its readers who may suffer from causes similar to those by which I was so long and so grievously afflicted.

"It is generally thought by persons in such circumstances, that their cases are singular and extraordinary; and pious sufferers almost universally ascribe their sorrows to the immediate hand of God, who, as they suppose, has withdrawn his favour from them, and has given them up to the sad consequences of their transgressions. They are also exceedingly prone to believe that their suffering is entirely mental and spiritual, and not at all the effect of bodily disease: while, in many instances, they suppose themselves to be acted upon by a satanic influence.

"Such notions greatly aggravate the anguish which they feel, and dispose them to despair of any permanent relief, either now or hereafter. The instance which I am relating will serve to show that these notions are for the most part either partially or entirely groundless; and that such sufferings are the effects of corporeal disease, and the disordered condition of the nervous constitution. Afflictions of this character, like all others to which mortals are liable, are indeed to be traced ultimately to the will and permission of God Almighty, who for purposes inscrutable by men, suffers them to befall even the wise and good, as well as those of different character. We may and must conclude, that neither good nor evil happen but by his appointment; but we have the surest ground on which to believe that no suffering to which we may be exposed, in the present life, furnishes an indication of God's displeasure in individual cases; and it is the peculiar glory of the religion of Christ, that no living man is warranted to despair of divine mercy and forgiveness, but on the contrary, however deplorable his condition may be, he has God's sure promise that he shall obtain favour if he seek it with sincerity, humility, and perseverance.

"What share in human sufferings of the kind in question is permitted to the invisible and implacable adversary of God and man, I shall not presume to define. Much of what is false and mischievous on this subject may readily be found; but while the fact of satanic agency in the affairs of mankind, is too strongly stated by the sacred writings to admit of question or disbelief, I know of no scriptural rule by the application of which the influence of such agency may be safely discriminated from the action of the mind itself. The only criterion by which the spiritual and heavenly agency exerted in the minds and hearts of true Christians can be determined, exists in the effects which it produces: where the fruits of the Spirit are found, there the presence of the Spirit is manifest; and where the works of darkness are, there we may be sure is the presence of the prince of darkness. In every possible case, one rule is laid down, and one assurance given, 'Resist the devil, and he will flee from you.'

"The most skilful physiologist is entirely ignorant of the manner in which our bodies and minds exert their mutual action on each other; all he knows is that such action takes place; it is therefore no wonder that we should be utterly unacquainted with the process by which spirits, either heavenly or infernal, exert their respective influences on the souls of men. It is no inconsiderable attainment in divine, as well as human philosophy, calmly to acquiesce within the limits which the feebleness of our faculties assigns to us; and which we can by no exertions pass beyond. How much sorrow should we avoid by such acquiescence! How much of what men call knowledge should we disallow under its guidance!"

Mr. Walford, with the view of mitigating his sufferings, retired after his daughter's death into the country. In describing his feelings at this period he thus writes:—

"You will be able to form some conception of the state in which I was, when I relate the occurrences of the day on which I left home, and arrived at my friend's abode. Everything was prepared for my journey on the preceding evening, and I retired to bed at my usual time, in as tranquil a state as could reasonably be expected in my circumstances. I slept quietly until about five in the morning, when I suddenly awoke, in a condition which I am unable to describe with any exactness. I seemed to myself to be environed by a dense and sulphureous fog or smoke, and was so overcome by horror as to exclaim aloud, that I was ruined and lost, though I had no conception of the cause that induced the frightful apprehension. I continued, however, to exclaim, when my wife, awakened by the outcry, earnestly asked what was the matter? For some time I could reply only by repeating that I was ruined for ever. At length she entreated me to rise and get ready for my journey, which I did, under the influence of these extraordinary and unaccountable feelings. The morning was very cold, which appeared to revive me, so that by the time I was dressed and ready to set out, I was a good deal relieved. I had to travel about eighty miles by coach, and though freed from the notion of being lost, I was during the day in a very excited, yet gloomy and wretched state. The meeting with my friend, and the soothing effect of his company and converse, stilled, in a considerable degree, my perturbed feelings, and I went to bed without any fear of not sleeping.

"I nevertheless passed a sleepless night, and during the twelve succeeding days and nights, in all thirteen, I did not gain a moment's sleep. My nerves seemed to be rigid, and at the utmost tension, and my feelings were hard and unimpressible. I tried the influence of opium one or two nights, in tolerably strong doses, but it produced no effect, and I used it no more. I fully expected I should lose my senses, as it seemed impossible for me to endure the suffering. But I afterwards learned, under the pressure of keener agonies, that no one can estimate the degree of anguish which it is impossible for him to sustain.

"As it was my intention to be absent from home not more than a fortnight, I went to my sleepless bed on the last night of my stay, with the forlorn hope of getting some portion of that soothing anodyne; and as I was to set out at four in the morning, I withdrew very early. No sleep or drowsiness came over me for two or three hours, when a violent palpitation of the heart banished all expectation of repose, and I desired an apothecary in the neighbourhood to be sent for. When he came, he made the inquiries usual on such occasions, and said he could discern no indications of disease, but, possibly, the liver might be affected; advised an application to a physician, as soon as I could, after my return home; he administered no medicine, but recommended a foot-bath, and left me. Happily the bath answered its intention, and I fell into a profound sleep. I was roused early in the morning, and began my journey homewards, though a good deal depressed.

"The weather was frosty and cold, but when I got out of the coach for breakfast, all my sorrows had vanished; my appetite was good, and my spirits were buoyant, and I got home with an expectation of better times. I spent, however, a sleepless night, though I felt somewhat better than was usual, and met my pupils at the proper time, in the lecture room. As I proceeded with the business, they discovered that I was in great distress, and implored me to desist. I complied, but was never again able to meet them. I had never made any complaints to them, but I learned, after my recovery, that they had long suspected some unknown and great distress was preying upon me. It was the practice of the College, for the whole family to assemble for devotional purposes morning and evening. The morning service was conducted by me, and that of the evening by the students in succession. My prayers, which were always dictated at the moment of delivery, unconsciously to myself led my pupils to this conclusion.

"In compliance with the advice I had received, I called on the day of my return upon a physician, a very kind and long well-known friend; he said then but little, which was chiefly to direct a dose of colocynth to be taken, and promised to visit me as soon as the operation of the colocynth should be ascertained. He came, and said he was quite sure the liver was in perfect health. Having been previously informed respecting my feelings and conceptions of myself, he assured me my complaint was unassailable by any medical treatment; that medical men were wholly ignorant of the causes that were concerned in the production of such maladies, and of any methods of cure. He strenuously advised the cessation of all mental exertion, with the utmost possible avoidance of every disquieting concern; entreated me to abstain entirely from opium, and to consult no medical practitioners, as they could do no good, and might do much injury. His decided opinion was, that the brain had been over-worked, and was now, as he said, taking its revenge by demanding rest. The performance of my duties at the College was now suspended, and, after more than a twelvemonth spent in the vain expectation on my own part, and on that of the supporters of the Institution, that I might resume them at no very distant period, I relinquished my office, and my residence in the College. I retired to a house in Hackney, in which, during the space of rather more than four years, I underwent horrors of which it is impossible for me to convey an adequate conception."

Here Mr. Walford says he would be inclined to bring his narrative to a conclusion; but he says he writes with a hope of affording solace and comfort to some fellow-sufferers into whose hands his memoir might haply fall. After stating that he was "induced to make trial of travelling, and visiting several distant places, as Brighton, Nottingham, Birmingham, &c.," he proceeds as follows:—

"Once I set out in company with two beloved friends, for the Lakes of Cumberland, and the southern parts of Scotland, but was unable, through the extreme agitation of both body and mind, to go beyond Northampton, whence I returned home in deep despair of finding relief by any such means. I was persuaded to try what daily short rides in an open carriage, driven by myself, and accompanied by my wife, would do for me. This I soon discontinued, as I became more and more averse to the persons whom we met, and the places through which we passed. I could scarcely endure the sight of strangers; and the visits of my friends, who called with the intention of consoling me, soon became so irksome as to induce me to secrete myself from them. Several pious friends proposed to me to permit them to hold a meeting for prayer with me, but the proposition excited my alarm to such a degree, that if they had not desisted I should have become frantic and violent.

"I began to shut myself up in solitude, as walking or riding through the streets made me feel as though every one I met was acquainted with my wickedness and misery. I could not endure to look anyone in the face, and ere long, the sight of my own face filled me with fear and aversion, as I considered myself to be wholly a reprobate, forsaken of God and odious to man. This unhappy sentiment originated in an irrepressible notion that I had been unfaithful in the performance of my duty, especially that which was connected with my college residence. Every instance of languor, deficiency, and imperfection which came to my remembrance, was so magnified and exaggerated as to appear of the most criminal and unpardonable nature. Before I left the College, I felt assured that I should not survive the day of its taking place, so that I looked forward to it with inexpressible dread and horror. The conception I entertained of my unfaithfulness became so powerful as to convince me that I had no sort of right to retain the property I possessed, and I even contemplated selling the stock which I had in the funds, that I might in some mode or other make away with it, though I was aware such a measure would reduce me and my family to absolute penury and want. The dread of negotiating this sale and making the transfer, which could not be done but in my presence at the Bank, deterred me; though I had so much power over myself as to execute a warrant, giving to my wife authority to receive the interest, lest in some reckless hour I should perpetrate so perilous a deed.

"My worthy and most sympathising friend, the Treasurer to the College, to whom I intimated what was passing in my bosom,—for strange as it may appear, I could not restrain myself from divulging nearly all my feelings,—used every expedient he could devise from day to day, to persuade me that all my misery originated in delusion, and that no greater satisfaction could be felt by all the patrons of the institution, than would result from my return to the office I had holden. But his endeavours were all fruitless, and I continued in hourly dread that I should be reduced to abject poverty, and end my days in a work-house, a prison, a lunatic asylum, or a ditch; and not improbably by my own hands. For many months I suffered from disordered action of the heart, and a remission of pulse, which, whenever I was excited,—and almost every occurrence produced excitement,—occasioned a species of convulsive action, which I thought would suffocate me. Besides which, I appeared to myself to be surrounded with a dense vapour, that prevented me from clearly beholding the objects of vision. My nights were often sleepless, and I was in such constant alarm and trepidation, that I could not allow myself to be left alone for an instant, without uttering cries of agony. In such a condition, a year slowly glided over me. I was not, indeed, at all times equally oppressed, as now and then, chiefly in the latter hours of the day, I was so freed from my gloom and dreary apprehensions, as to feel some measure of cheerfulness, which tempted me to hope for entire deliverance from my grievous bondage; but after many alternations of such feelings, I learned that no reliance on the flattering hope could be exerted, as in a few hours the brightness vanished, and the clouds accumulated as thickly as ever. The morning hours were invariably the worst seasons of the day.

"After the expiration of this first year, all my distressing symptoms increased in strength and continuance: the remissions of which I have just spoken, became less and less frequent; and during the succeeding four years, I was oppressed by unbroken darkness, and tortured by anguish, which I will describe as well as I am able in my next letter, though no words can express with adequate force the terrors through which I passed.

"I am quite at a loss to relate in the order of their occurrence, the truly frightful sufferings to which I was subjected; nor is it either possible or desirable I should recite the half of them. In the course of the first year to which I have adverted, I was disposed incessantly to talk of my feelings, and to weary the members of my family by reiterated complaints. I had habitually no religious feelings, but such as were made up of the keenest anguish, on account of the loss of all those pleasures which I had formerly enjoyed, in exercises of public and private devotion, and of the utter despair in which I was involved, of obtaining the future blessedness which is promised to all the faithful disciples of our Lord Jesus Christ. Sometimes, however, during that period, an unusual excitement to pray would so prevail over me, as to induce me to desire all who might happen to be in the room where I was, in whatever they might be engaged, to kneel while I addressed supplications to Heaven, with an earnestness almost frantic, for some alleviation of my intolerable anguish. After about the period of which I write, these impulses altogether forsook me, and I for days and weeks together used no prayer, unless that now and then a passionate ejaculation would escape from me.

"I now shut myself as much as possible from the observation of any but my own family, and for two or three years never passed the threshold of the street-door. I abandoned all public and social devotion, as I could not bear it; and thought it vain and useless for my condition, which I felt assured was that of a lost and reprobate wretch. Not unfrequently when called to dinner, I rushed out of the house into the garden, because I could not dare to implore a blessing, or express any thankfulness to God, who had, as I believed, entirely and finally deserted me, and had become my Almighty enemy. Books of every description I ordered to be removed out of my notice, and insisted on the whole of my library being sold, at whatever loss might be incurred, and that was considerable, as I had paid exorbitant prices, on account of the closing of the continent during the French war, for a large number of them, and which were extremely depreciated by the return of peace, which opened a free intercourse with all parts of the world.

"My reason for this procedure was, that books of every kind, especially religious ones, and the Bible in the greatest degree, were associated with remembrances that I would gladly have banished for ever from my mind. I earnestly wished I had never learned to read or write, while at the same time I felt the strongest desire to engage in both, but was driven from them by the morbid sensibility which was so extreme as to be affected by every topic of thought that was presented to me. Similar feelings constrained me to shun the converse of my friends, though I was passionately desirous of their converse. I could compare myself only to a human body, the skin of which having been stripped off, no part can be touched without inflicting agony. This condition at length increased to such a degree, that I could not bear the ordinary conversation of the members of my family, whether they were sad or cheerful. The light of day so distressed me, that I had all my windows blinded: the sun, the moon, and stars filled me with inexpressible dread, and I beheld them as seldom as was possible. All ornamental furniture, especially looking-glasses, was especially offensive to me, and was removed from the apartment in which I lived. My own personal appearance was neglected to the utmost; I should never have shaved myself, or changed my clothing, but for the affectionate remonstrances of my wife; nor could I endure the thought of having new clothes made. For what purpose, I said to myself, should an outcast wretch like me pay any regard to external appearance or ornament? It seemed even shocking to me, and monstrous.

"My irritability of temper was so great, that I fully expected, in some fit of passion, I should murder some of the inmates of my house; and this notion became so strong, that for about two years it was seldom absent from my thoughts; so that I often, in imagination, underwent all the forms of public prosecution; invented speeches I would make at my trial, when I knew I should plead guilty; and endured agonies in this way that could scarcely have been exceeded by reality. During the last four years of my extraordinary wretchedness, I was perpetually haunted by an extreme apprehension that I should destroy myself, in order to get free from the incessant torment I was compelled to endure. I never indeed proceeded to any actual attempt on my life, though I was very often revolving the different methods of destroying it, and considering which I should choose. My patient wife was sometimes cautioned by her friends to remove from me, as far as possible, the means of destruction; and I was continually telling her of the thoughts that were perpetually present to me. Often I asked if she was not afraid of living with me? but her reply invariably was, 'Not in the least degree.' She knew me too well, and was too confident of God's mercy to herself and to me, she said, to have any such apprehensions.

"The agitation and restlessness that affected me were so great, that I was unable to sit down, as the moment in which I attempted to do so brought an increase of misery; and I was thus kept pacing up and down my parlour from the time of getting up until going to bed. I was so intensely wearied by this incessant going to and fro, as frequently to scream with anguish. In consequence of this painful excitement, I seldom rose from my bed before noon, as I was able to continue this posture without additional pain. As soon as I came down stairs, I hastily swallowed my breakfast, standing, and then the endless movement began. While my body was thus occupied, my mind was the seat of the direst contemplations, revolving the past and the future, until sometimes, when thinking of my pious friends who were no longer living on earth, I loudly bid them an everlasting adieu, as I was never to be admitted to the rest to which they had been conducted, or join in those strains of celestial harmony that resound through the abodes of the blessed and immortal inhabitants, and to which I formerly hoped I should be conducted, when the trial of life should have been surmounted. On such occasions, sighs of distress, so deep from my bosom, would involuntarily escape, as too plainly indicated the profound sorrow that affected me. To this day more than twenty years have passed away, yet I am often surprised by sudden sighing, which, though unassociated with any sentiment of distress, occasions a temporary emotion.

"Through this weary, toilsome, and excruciating period, my nights were often almost if not quite sleepless. When endeavouring to compose myself to rest, I was often roused to vigilance by convulsive startings, which no sooner ceased, than the most hideous appearances of monstrous face and shape would pass before me, to free myself from which, I was constrained to keep my eyes open, that the real objects about me might dispel those of my disordered imagination. How often did I exclaim, in the words of the suffering patriarch: 'The arrows of the Almighty are within me, the poison whereof drinketh up my spirit. . . . . When I say, My bed shall comfort me, my couch shall ease my complaint; then thou scarest me with dreams, and terrifiest me through visions. . . . . Thou writest bitter things against me, and makest me to possess the iniquities of my youth.' Amidst these bitter agonies, I was annoyed more than can be imagined by a cause which seems trivial, but was far otherwise. Very often persons, places, and things, would occur to me, the names and particular appearances of which I was unable to recall without long endeavour of a most wearisome kind. I could not remember the name of some one, nor present to my fancy the faces or forms of various persons or things with which I had been familiar; nor could I banish them from my thoughts, but was constrained to use every method I could devise to bring to my remembrance what I was forced to pursue, until I alighted on the name or object that was suggested to me. Days together was I employed in this fruitless pursuit, without being able to discover what I wanted. Often when found, it would suggest to me something else of the same kind, with similar disquietude, till I felt that the labours of Sisyphus were less fatiguing and useless than those from which I could not escape. My nights were often greatly disturbed by the sudden occurrence of some such things, that suddenly darted on me when nearly asleep, and instantly banished all power of sleep.

"Early in the commencement of the four years to which I am directing attention, a suggestion was made to me by some friend to try some game, as chess, bagatelle, drafts, &c. At first, I rejected the proposal with scorn; but on afterwards conceiving a hope that, perhaps, somewhat of this kind would enable me to sit so much as to avoid the extreme weariness of being always on my feet, I made trial of chess, which succeeded so far as to keep me on a chair. Having made this discovery, I called regularly for the chess-board as soon as I came down stairs in the morning, and insisted that my wife or niece (who lived with us) should play the whole day, until it was time to retire for sleep. In this manner I played thousands of games, sometimes varying the employment by backgammon, &c. Some inconsiderable degree of alleviation was thus obtained during the day. As, however, my companions were compelled at bedtime to withdraw, I was then left alone for hours, as I exceedingly dreaded to go to bed, on account of the sleeplessness and other innumerable disquietudes which I almost invariably had to encounter. I therefore paced up and down the room until midnight, or later. These solitary hours were the most afflicted I was doomed to suffer. Sometimes I was so alarmed lest the anger of God should suddenly fall upon me, and seal my doom, that my limbs trembled with the agitation of my thoughts.

"It would not be difficult to enlarge this relation of misery, but enough has been said to weary me in the recital, and more than enough, I fear, to weary the reader of these dreary pages; I shall, therefore, after observing that these midnight hours were rendered, in some degree, more easy by my finding out that I could play backgammon without a partner, as the game very much depends on the dice, terminate the harrowing detail.

"I must, however, remark that every hope of recovery was long banished from me, and I believe from my friends: this utter prostration of hope aggravated, as much as aggravation was possible, the misery of my condition. Yet recovery at length, though long despaired of, came, and through the great mercy of God, I was rescued from 'the horrible pit, and the miry clay,' into which I had seemed to be rapidly sinking."

This state of mental gloom and despondency was, however, to come to an end. We will quote in detail Mr. Walford's own account of his extraordinary recovery:—

"The blissful recovery which I experienced was not to be ascribed to any medical process whatever. I had, indeed, much against my own inclination, been so importuned by my friends as to consent, three or four years before my recovery took place, to consult one or two medical advisers; but the effect proved, as I fully expected, that nothing was to be hoped for from this expedient, and I positively refused to see any other medical persons. About the same time, I was over-persuaded, on account of my general inability to sleep, to keep laudanum by my bed-side, and to have recourse to it when sleep was found to be impracticable. I tried this measure two or three times without any sensible effect, and firmly resolved to take no more. I adhered to my purpose, and no other experiments of the kind were ever adopted. A few months before any symptoms of improvement appeared, I now and then prevailed on myself to walk up and down a few hundred yards in the road adjacent to my house, when I was concealed by the darkness of the night from the notice of any who might pass me. Soon after, I went several evenings, when the light of day had departed, into my garden, and paced up and down for some time. On these occasions, I sometimes felt an impulse, during my walks, to pray with deep fervency, that some measure of relief might be afforded to me. These prayers were short and broken, yet I trust they found acceptance in heaven.

"Some weeks or months after these occurrences, an old friend from Suffolk, a most worthy minister, came to see me, and stayed a day or two. I had formerly smoked many a pipe of tobacco in company with my friend, though for the preceding five years I could not bear the sight of a pipe. My wife, aware of his habits, had the materials for smoking set before him, which he employed, and earnestly pressed me to accompany him, which I passionately refused to do. On the evening of his departure, when, as usual, I was the only person sitting up, it occurred to me to try if I could smoke, which four or five years I had discontinued, on account of the manifest bad effects which it produced on my pulse: I instantly procured for myself the smoking apparatus, and found I could perform the operation without the injurious results which had induced me to relinquish the practice. Soon after this experiment, I resolved to try if I could read, though I was under a great difficulty to select a book that did not seem likely to awaken painful associations, and I especially shunned all such as treated of religious subjects. Accident determined my choice. I had not relinquished a Book Society of which I was a member, though the books that came to my house were carefully concealed from my notice. At the time of which I am now writing, I found that a 'History of the Cotton Manufacture,' by Mr. Baines, was brought to my house, and as it seemed not very likely that anything in it would excite my feelings, I resolved, though with extreme apprehension, to try this book. In a day or two, I found nothing in it that much distressed me, and I perused it to its close. It amused me, and after reading it again, I wrote out a pretty extensive abridgment of it. I then attempted a work by Mr. Babbage, the title of which is, I think, 'The Economy of Manufactures.'

"After reading and epitomising these works, I was so much quieted as to regret I had no others of similar character: and I then engaged in writing a translation of the history of Herodotus. Before I had completed my translation of the first book of that history, the spring brought the month of May. My son entreated his mother to take a ride in a carriage with him, and I joined in the entreaty, as I greatly wished she should enjoy some refreshment of this kind. The carriage was brought to the door, when my faithful wife positively refused to go unless I would accompany them. This, I both thought and said, was impossible. She, however, persisted in her refusal; and for some time I warmly remonstrated with her, and urged her going. While I was thus engaged, a sudden inquiry offered itself to me: Why I could not go? I could discover no reason; and calling for my hat, I jumped into the carriage, when I directed the driver to take us to Epping Forest, through Wanstead and Woodford, a ride which, in former years, I had often taken with great pleasure. The verdure of the grass, trees, and country in general, with the fineness of the weather, so affected me, that all my fears, disquietudes, and sorrows vanished as if by a miracle, and I was well,—entirely relieved, and filled with a transport of delight such as I had never before experienced. My hope and confidence in God were restored, and all my dreary expectations of destroying myself or others were entirely forgotten. On my return home from this reviving excursion, every desire to shut myself up and exclude my friends was departed, and I could with difficulty restrain myself from being always abroad.

"This extraordinary change of feeling took place, as I have said, in May; and on the first day of the following August, I set out in company with my son and an active friend, who had before travelled on the continent, for France, Switzerland, and Germany. The delights of that journey were so enhanced by contrast with the events of the five preceding years, that I was in a species of rapture throughout the whole. I felt no apprehensions of danger in going so far from home; and the glorious scenes I witnessed so enchanted me, that my pleasure overflowed the limits of ordinary enjoyment. One only regret was occasioned by the unavoidable necessity, under which my companions in travel were placed, of returning at the end of the month to business; by which I was hurried from scenes of surpassing grandeur and interest, before I had half gratified myself with gazing upon them. Enchanted and fascinated as I was with this tour, I attribute no part of my recovery to it, as I had been entirely freed from my sad condition, both of body and mind, before it took place; if this had not been the case, no wishes of my own, nor any entreaties of my friends, would have had power to persuade me to set out upon it, so deeply was I affected by the remembrance of former disappointments. Immediately after my return, I was seized with a most unexpected and severe diarrhœa, which I thought would terminate my joys and sorrows alike: it yielded, however, to skilful medical treatment, after some days; and one of my medical attendants, who had long been acquainted with my constitution, assured me when the vehemence of the paroxysms was abated, that the effects of it were far more beneficial than any medical treatment could have produced; and he anticipated a perfect freedom from the return of my distressing nervous disease. This anticipation has been verified by several successive years of established health; and though I am now occasionally in some measure disturbed by some of the minor symptoms of my disorder, for short periods, chiefly during the hours of night, my general health is remarkable for my years; and the condition of my feelings tranquil and cheerful, though seldom much elevated.

"It will not appear surprising that, after the singular and remarkable detail that has been given, I felt a great wish, if possible, to ascertain the cause of nearly fifty years' intense suffering through which I had passed. While, indeed, under the great force of my sorrows, I was evermore induced to regard them as arising from mental and spiritual causes, quite independent of any bodily disease; yet as soon as my recovery was confirmed, I strongly suspected this notion was incorrect, and some recollections of former years led me to the conclusion that it was quite unfounded. My natural temperament had ever appeared to me to be more of the sanguine than the saturnine species, so that whenever I possessed myself sufficiently to consider the case impartially, I imagined that somewhat superinduced, and not native, was the source of my melancholy despondency. I shall therefore briefly mention the occurrences that befell me, and the conclusion from them at which I arrived, by putting them together. My knowledge of physiology is very scanty, so that if my memoir should meet the eye of any proficient in that science, I must crave his candour for what I am about to state relative to my belief of the nature and causes of my complaints. I have what appears to me to be a probable judgment on the whole case, which, however, I do not presume to represent as if it were demonstrated, or as entitling me to impose it on persons who are alone qualified scientifically and religiously to decide respecting it.

"From a very early age—say five or six years from my birth—I was subject to very frequent attacks of headache, which increased in intensity up to about the twenty-second or twenty-third year of my age. At this time I was a student, and my studies were very often seriously affected by this frequency of pain. About this period, my attention was forcibly drawn to an increasing discharge of fetid mucus from one (the left) of my nostrils, which became very offensive to me, and clearly indicated something wrong on the left side of the upper part of the interior of my head. The first surgeon in London was at that time Mr. Cline, to whom I applied for advice. His opinion was that an ulcer had formed in the frontal sinus, which he supposed to be easily curable could it be got at: as this was impracticable, he recommended certain applications of mercury to the nostrils, the vapours of which were to be conveyed by the agency of heat into the head. This practice was tried for some time, but served only to aggravate the symptoms, and was therefore discontinued. Soon after this experiment, I went to Birmingham to visit my friends, when it occurred to me to consult Dr. Withering, who was then practising as a physician with eminent reputation. He did not coincide with Mr. Cline's opinion, but inquired if I had at any time suffered an injury upon the skull? It had never before occurred to me to reflect on what I had often heard from my mother, that when I was about two years old I had fallen on the edge of a fender, and inflicted a very dangerous wound on my forehead, the scar of which was at that time quite visible. On directing his attention to this mark, he instantly said, there was the origin of my pain; a wound had been produced in the interior of the sinus, which he feared would never be healed, though it was not impossible it might be worn out by the increase of years. He cautioned me against allowing any tampering with it, as it was impossible to do good, and injury might be inflicted. He advised the application of leeches, whenever the pain might be very troublesome; and recommended me to take snuff plentifully, as the means of stimulating the secretion of mucus, in order to assist the escape of the purulent matter that was ever forming, and was the chief cause of the headaches from which I suffered.

"This advice appeared to me to be wise and good, and I immediately acted upon it, with great ultimate though not immediate benefit, as my headaches continued without material diminution for perhaps four or five years. Soon after I went to reside in Yarmouth, which was when I was on the point of thirty years of age, I experienced the first serious attacks of the malady, the growth and termination of which have been described in the last letters, and need not therefore be repeated. After the first of these paroxysms of despondency took place, I gradually perceived the headaches by which I had been so long afflicted were almost imperceptibly becoming less frequent and intense, while the symptoms of dejection increased in about the same proportion. These changes were so slowly effected as to elude much observation at the time of their occurrence, though I subsequently became painfully conscious of them, in the great increase of my mental suffering, and the almost entire cessation of the hemiplegia, or partially local headache. After my recovery, when often reflecting on the course of suffering through which I had passed, it occurred to me that the headaches and the mental depressions were the results of one common cause—the injury inflicted on my head in infancy. I conceived that the headaches were the effects of the injury, so long as the consequences of it were confined to the sinus exterior to the brain; and that the mental suffering, which by slow degrees succeeded to the decreasing pains of the head, and finally displaced them, was caused by what medical men technically term *metastasis*, or a transference of the effects of the injury from the external sinus to the interior of the cranium, and these so affected the brain and the nervous system that is dependent on it, as to produce the grievous sorrows of which I was for almost sixty years the subject.

"The conception now stated is entirely my own, and if it be incorrect, the incorrectness belongs only to myself, and I have set it down as a probable reason for attributing many of what are called nervous diseases to injuries immediately or remotely affecting the brain and the nervous system, in a greater or less degree, though I hope and believe, in few instances so pregnant with intense and durable wretchedness as that from which I was mercifully delivered. My notion, which I have briefly developed, of the origin of my malady, derives some confirmation from the observation made to me by Dr. Withering, which I have noticed, that possibly the effect of my early injury might be outgrown by advancing years. Such, I imagine, is the probable reason of my sudden and almost instant recovery: the cause was exhausted, and the effect ceased."

When referring in another portion of his autobiography to his restoration to health, Mr. Walford, when speaking of the probable physical cause of his attack of mental depression, observes:—

"Ignorant, prejudiced, and irreligious men are frequently guilty of ascribing such a derangement as that which I have described, to false conceptions of the nature of religion, and the extravagances of heated and fanatical imaginations—the results of puritanical or methodistic representations of Christianity. By such means they endeavour to discredit all serious and spiritual piety, and to justify their own careless and wicked disregard of it. In the instance which this memoir offers to observation, it is plain and undeniable that the dejection, melancholy, and excited apprehensions of misery, present and future, would have agitated any individual whatever, religious or irreligious, who should have suffered from physical injury a similar disturbance of nervous and mental health. The specific objects on which the morbid influence is exerted will vary, according to the several predominant characteristics of the persons affected by it: irreligious men are as liable to such injuries, and the natural effects of them, as any of directly opposite and contrary character; but the special effects will differ, just as the individual habits, mental and moral, differ from each other. A bad man may be the victim of nervous derangement, but his dejection will not be associated with anguish arising from his apprehension that he has lost the favour of God, together with the forfeiture of all the pure and heavenly delights which his dependence on God, and his converse with him, were wont to impart: his distress may be very great, but it will have no relation to his exclusion from the 'spirits of just men made perfect,' and a final separation from his friends whom he had loved on account of their sympathy in holy affections, and in converse with whom he had been expecting a friendship more perfect and an intercourse more blissful than can be enjoyed in this mortal and transient state. Other fears, apprehensions, and terrors will engage his thoughts and agitate his bosom, according to the strength and virulence of his perturbed imagination, but they cannot be imputed to either his supposed or actual piety, as he is possessed of neither."

Mr. Walford's recovery was not, however, a permanent one. The Rev. Mr. Stoughton, in his continuation of the memoir, says:—

"On the 21st of December, 1849, the Editor received from his revered and beloved friend a note—the last he ever sent to him—containing an invitation in the following terms, which show the feelings with which he anticipated the birthday that proved his last:—

"'If I am permitted to live until the 9th of January next, I shall have completed my seventy-seventh year; and I write for the purpose of saying that I hope you and Mrs. Stoughton will come and dine with me on that day, and spend as many more days and nights with us as you can afford. You will not, I hope, allow anything, if possible, to prevent my having this pleasure, as I cannot look for returns of that day without presumption.'

"The invitation was gladly accepted, and the day thus spent with him was one of unusual gratification. Infirmity seemed to have but slightly touched his vigorous constitution, while age had not at all impaired the energy of his strong intellect, or cooled the ardour of his domestic and social affections. His conversation was, as ever, intelligent and sensible, and indicated that his mind was in a state of placid enjoyment. It was interesting to see his manly and venerable form, seated at the fireside of his library, surrounded by his favourite authors, to listen to reminiscences of old times, and to remarks, pronounced in no undecided tone, on various topics, theological and literary, and to witness the beaming forth of that unaffectedly genial spirit which always crowned his simple, but generous and hearty hospitalities. Old age with a matured mind and a mellowed heart is always beautiful. It resembles a tree tinted with autumnal hues of glorious richness, and reflecting from its leaves the brilliant rays of sunset. A charm of precious holy power invests it, which whoso feeleth not hath a dry and withered soul. The subject of these recollections was a choice specimen of such old age, and rarely was it seen in a better light than on that last birthday.

"A few weeks after this, intelligence of an alarming kind respecting him was received by his friends in town. He had become seriously indisposed, and in connexion with very considerable physical disease, some plain symptoms of his old melancholy had made their appearance. But after a little while there was a marked improvement. Health, bodily and mental, seemed as if they would be entirely restored. During a visit paid to him by the writer, Mr. Walford expressed the alarming apprehensions he had felt lest his previous sad visitation should return in unmitigated force. And then with touching simplicity, while his eyes were full of tears, and his lips quivered with irrepressible emotion, he adverted to the efforts he had used to quell his dark forebodings by a humble and believing application of the Gospel to his own case.

"'I can only rest,' said he, 'on the most general assurances of the divine mercy; declarations which include all are alone such as I can believe include me. 'If *any man* sin, we have an advocate with the Father, Jesus Christ, the righteous: and he is the propitiation for our sins: and not for ours only, but also for the sins of the whole world.' That is *general* enough. It gives me hope.'

"After repeated references to this encouraging topic, he resumed his wonted love of conversation, discussing several theological and critical questions with unabated interest; and as some references happened to be made to popular methods of representing truth by the aid of imagery and illustration, he observed that such modes, though at times very important, desirable, and even necessary, could never yield satisfaction to a mind of his habits, desiring as he did to get below the outward covering and drapery of truth, to investigate its essential nature, and to form clear, definite, and abstract views of its substance and soul.

"The impression left by the interview was highly encouraging, and it was fervently hoped that the last clouds of eventide had broken and would vanish, and that the going down of the sun would be a scene of glory. But the hope was soon checked. Tidings of relapse, fearful relapse, succeeded. Another visit was paid, and how different was the scene in the quiet little study at Uxbridge from that witnessed only a few weeks before! How very different from the cheerful birthday scene! Disease had manifestly been at work. The form had shrunk; the face was haggard; the sunken eye indicated despondency. He made an affectionate acknowledgment of his friend's kindness in coming to visit him, but expressed himself as no longer entitled to the pleasures of friendship, no longer worthy of esteem and regard. Books, which he had so much valued, he declared were now a torment to his sight. Philosophy, his favourite pursuit, he denounced as a word he could not endure. Incessant restlessness would not allow him to remain in his chair for a minute, but he was ever pacing his study with perturbed emotion. It was night, dark, starless night, with that soul that had once been so bright and sunny. All efforts made to administer consolation were instantly repelled, and he dwelt with agonizing earnestness upon his state of mind, which he described in terms characteristic of his usual correct and exact habit of expression, but swelling out sometimes into bursts of unwonted eloquence—the eloquence of despair. It was plain that with the recurrence of physical disease had come the dense morbid melancholy of bygone years.

"It was a painful task for those who watched him to see with what power the malady operated on his mind; not merely beclouding his thoughts with regard to religion, but, as in the former case, producing strange ideas and fears with regard to his circumstances. Yet, amidst his mysterious hallucinations there came now and then, especially once, a lucid space in which disease gave way, and Christian faith and hope burst forth."

Continuing the narrative, his biographer observes:—

"A third visit paid by the writer was scarcely less mournful than the second. The bed-room, to which disease and infirmity now confined Mr. Walford, so darkened, by his express direction, as scarcely to leave his features visible, was but as a sign and symbol of the mental state of the venerated and much-loved sufferer. With perverse acuteness he parried off all arguments of consolation, and obstinately averred that while his distress twenty years before was the effect of disease, his present sorrow was independent of such a cause. His explanations of the former visitation were repeated, but in vain. His pertinacious refusal of all comfort was, however, but too plain a proof of the renewed and entire ascendancy of that same insidious morbid influence which had previously been such a destroyer of his peace. Still it was hoped that a time of joy would return. Anxiously did his affectionate niece, who resided with him, and his not less affectionate daughter-in-law, who spent the last few weeks under his roof, watch and wait for such a happy season, even as the watchman waiteth for the morning: but in this world it never came. The paroxysms of anguish, indeed, abated; he spoke less and less of his sorrow, and sunk down into a state of silence, if not torpor. Days and nights gloomily rolled on, so different from their 'tranquil gliding' which he frequently described in his letters and other papers; but it was the happy confidence of his friends, notwithstanding his own fears, that the angry billow, no less than the gentle wave, was bearing his weather-beaten bark to the better land. That land he reached on the 22nd of June, 1850. The poor body looked truly like a wreck; but the eye of Christian faith could see the soul, which had often had such hard work to pilot the unmanageable vessel, safe beyond the reach of storms and the return of night, on the shores of the heavenly country."

We copy for the perusal of those who are interested in this case, the account given in the appendix of the *post mortem* examination of Mr. Walford. It is as follows:—

"Examination of the body of the Rev. William Walford, on the 27th June, 1850, the fifth day after his decease:—

"No remarkable external appearance; there was more fat over the whole body than could have been expected, when his long illness and great abstinence from food are considered. On opening the head, the dura mater was found so firmly attached to the bone at two points, as to be incapable of separation without being torn. Those two points were—one near the superior and anterior angle of the right parietal bone, the other at the superior and posterior angle of the left parietal bone: they were marked on the internal surface of the bones by deep depressions having a sort of honeycombed appearance, but not carious. The outer table of the skull alone remained at these parts, and its thickness scarcely exceeded stout letter-paper; the size of both depressions was nearly the same—about an inch long by three-fourths of an inch in breadth. The colour of the brain under the first point was different from all its surrounding surface; it had assumed a green tinge similar to long-retained pus: this did not extend more than a quarter of an inch into the substance of the brain. There was no discoloration of the brain at the second point, nor was there elevation of the surface at either: the depressions in the bone were from thickening of the dura mater in those specified localities. The dura mater throughout its whole extent had lost much of its proper vascularity, and assumed a thickened yellow, leathery appearance. Over the whole surface of the brain there was considerable serous effusion: the ventricles were full of water—there were no signs of recent inflammatory action, but there were several points of unnatural adhesion of the membranes, denoting former existence of an inflammatory state. The lungs were sound throughout, but had large adhesive bands at various parts, the consequence of inflammation at some remote time. There were several ounces of water in both sides of the chest.

"The heart was large, flabby, and covered with a good deal of fat, especially at the base. It contained no blood—it was strongly adherent to the pericardium over the whole space corresponding to the left ventricle, the evident effect of inflammation at some former time. The valves of the heart were sound; the aorta was fully one-half larger than natural, and at its origin from the heart, was an almost continuous circle of ossification. The whole inner surface of the left ventricle and of the arch of the aorta had a deep red colour, like inflammation, but there were no enlarged capillary vessels to be seen. The pericardium contained about an ounce of water. All the abdominal viscera were in a healthy condition.

"DAN. MACNAMARA, Surgeon,

WILLIAM RAYNER, Surgeon, } Uxbridge."

We now proceed to direct the attention of our readers to another work presenting many points of deep psychological interest. In the memoir of Mr. Richard Williams, surgeon, who officiated as catechist to the Patagonian Missionary Society in Tierra Del Fuego, we have the details of an extraordinary mental attack, during which this gentleman is said to "have undergone marked spiritual changes." We subjoin Mr. Williams' narrative of the singular illness which issued in what is termed, "his conversion."

"I bless God that ever I was afflicted. Not only do I date my conversion from my illness, but I believe that this illness was designed for my conversion. It was a seizure more remarkable than any of which I remember to have heard or read; and, apart from the inward working of the mind, it presented a series of extraordinary symptoms, which seem to defy solution. Myself a medical man, and for many years accustomed to witness disease in every form, I have been able to explain, to some extent at least, almost every case; but for the cause of my own illness, and for the explanation of its strange symptoms, my knowledge and means of judging fall far short. But whether mere natural causes occasioned all the bodily sensations or not, scarcely signifies: the mental changes, I am fully assured, were altogether the work of God.

"At the very outset, I should acknowledge that I had no previous belief in the truth of Christianity. I viewed it sometimes in one light, sometimes in another. I regarded it, for the most part, as an absurdity. At its many votaries I wondered, and their understandings I looked down upon as strangely deluded. I could not comprehend how a God should die, nor even bring my mind to admit that an atonement was necessary. The works of infidels, however, I always read with dissatisfaction or disgust; and any scurrilous attack on the faith of others I should have been ready to oppose. But into the truth of the matter I never thought of inquiring; and, as far as my perusal of it went, the Bible was a mere lumber-book. Science, literature, and my profession, were my whole delight; but the truth or falsehood of Christianity I felt it no part of my business to examine.

"Of natural religion I had something in my heart. Many a time have I lifted my eyes from nature up to nature's God, and have adored his excellency as revealed in his beautiful and magnificent works. I knew myself to be a creature sprung from God; but I never dreamed that I was a creature accursed before him. I knew God to be infinitely just; but I never feared that that justice would consign me to eternal misery. I knew that I oftentimes acted contrary to my conscience; but I believed that intellectual enlightenment and the mere force of reasoning could carry human nature to perfection, and place it far above the control of passion. I deified human nature as capable of transcendent virtue, and absolutely denied its innate corruption. I hoped that the soul was immortal, but could never feel convinced that it was so; but as to everlasting torments,—I viewed the doctrine as sacrilege, and a defamation of the justice of God. The existence of a devil I believed no more than any other bugbear.

"The only instances when confidence in my own opinions has been altogether shaken, were, I well remember, moments when, without an assignable reason, I have awakened from sleep, and an indescribable awe and terror have seized on my soul, filling it with undefined apprehensions of the future.*

"Such is a slight picture of my state of mind previous to my illness.

* "To such lucid moments does Jane Taylor refer, in lines not the less poetical because of their simple truthfulness:—

'And yet, amid the hurry, toil, and strife,
The claims, the urgencies, the whirl of life,—
The soul—perhaps in silence of the night—
Has flashes, transient intervals of light;
When things to come, without a shade of doubt,
In terrible reality stand out.
Those lucid moments suddenly present
A glance of truth, as though the heavens were rent;
And through that chasm of pure celestial light,
The future breaks upon the startled sight;
Life's vain pursuits, and Time's advancing pace,
Appear with death-bed clearness, face to face;
And Immortality's expanse sublime,
In just proportion to the speck of time:
While death, uprising from the silent shades,
Shews his dark outline ere the vision fades;
In strong relief against the blazing sky
Appears the shadow as it passes by.
And though o'erwhelming to the dazzled brain,
*These* are the moments when the mind is sane;
For then, a hope in heaven—the Saviour's cross,
Seem what they are, and all things else but dross.'

*Essays in Rhyme.*"

Up to the moment when it seized me, I had been engaged in the active duties of my profession. I had visited many patients, and during the evening had felt fatigued and languid, and anxious to seat myself comfortably in my arm-chair. A little after ten o'clock I saw the last of the persons waiting for me, and instantly I felt myself severely unwell. I went up-stairs, and threw myself on my bed. In a few minutes I felt inexpressibly ill. The first sensation was an amazing weight on the chest, with difficulty of respiration; the carotids of my throat striking like hammers on my head, and a feeling as though torrents of air were rushing into my brain, and the head were itself expanding. The agony became insupportable, and I knocked for some one to come to me. Meanwhile my mind acquired a wonderful vivacity. Thought upon thought came pouring in with a distinctness of apprehension, enlargement of view, and faithfulness of memory, such as I never before experienced. A power to comprehend my personal identity, and to understand my relation to time and eternity, was wonderfully given me. The passing moment seemed without beginning or end. I felt as though immortal faculties, immortal relations, were beginning to be recognised. The thought began to stagger me, that the hand of death was grasping the cords of life. With the thought, darkness—thick, palpable darkness—gathered on my soul. A mountain load seemed to crush my breast. It was girt as with bands of iron. My heart felt too big for its wonted space. A horror of anguish filled my whole being. Unnumbered sins sprang up before my astonished conscience, and Death in his terror rose up to my gaze. Look where I would, there was no hope. One wide, unbounded ocean of dismay and terror, lashed with tempestuous howlings, roared on every side; and the thought of an offended God pierced my soul with madness and despair.

"In this state I lay for hours. Meanwhile my sister, alarmed by my knocking, had come and found me speechless. Others of my friends were sent for; then medical attendance. Recourse was had to remedial measures; but I still grew worse. The night passed, and the morning found me the same. A painfully vivid consciousness of everything going on around me added greatly to my distress. The first faint glimmer of light that broke into my soul was when the name of Jesus was uttered. With the very thought of that name the hope of mercy was allied, and like a drowning man I clung to that hope. In the agony of my soul I called upon that name; and in the meanwhile, finding that one of God's servants (Mr. M., senior) had entered the room, I felt a new hope, as if the very presence of a man of God was a source of safety. He bade me look to Jesus. With the very bidding I felt an infinite joy in so doing. Faith in that holy name rapidly gained the ascendant. My darkness was turned into light, and in a short time I felt a sweet sense of the pardoning mercy of God. After this I grew better and better, and all my symptoms remitted, 'till I felt nothing except the languor resulting from the violence of my previous sufferings.

"Towards the evening, however, a relapse took place, with phenomena essentially different. Beginning with the same contraction of the chest as before, there followed tetanic spasms—a violent jerking of the upper part of the body from side to side, interrupted by quiet intervals, sometimes by a complete rigidity of the neck and spine. So sensitive was I to touch, or to the impression of a breath of air, that the approach of any one evincing an intention to disturb me would throw me into convulsions; and, suspecting tetanus or hydrophobia, the three medical attendants inquired whether I had been bitten by a dog, or had sustained any mechanical injury. With short intermissions, this state of things lasted for successive days, till my strength was nearly exhausted. Towards the close of the fourth day, and during the succeeding night, my eyes were upturned in their sockets; I retained not the slightest power of voluntary breathing; I was incapable of speech; and the attempt to swallow a drop of water brought on spasms which threatened suffocation.

"During all this period I was possessed of perfect consciousness; nor had I any pain. The only painful sensation was the impossibility of resisting the convulsive movements of my body, and the fearful constriction of my chest. At first I was, as it were, a mere spectator and observer of the symptoms—thinking, and even reasoning upon them; and when abstracted from their consideration, I felt that I could calmly meditate on God's mercies. I had no painful conflicts about my state, but a settled serenity—a tranquillity for which I could scarcely account, unless from the conviction that my salvation was sure. But during the last night of this stage, I experienced wonderful evidence of a world to come. My friends were assembled at various distances around my bed. The curtains were drawn, and a candle yielded its obscure rays. I heard the sobbings of my relations. I knew that they looked on my life as fast fleeting. I was myself convinced that I should not recover. I had pictured my body carried to the grave, and had marked in my mind's eye all the attendant circumstances. Mentally I had taken leave of earth, and I lay in perfect peace, assured of my salvation. A dead silence now reigned around; and as I waited the moment of my final change, it was an intense and deeply absorbing thought that soon the great scene would be revealed. Whilst lying thus, I thought I heard a gentle knocking. My soul started in expectation. Inwardly I exclaimed, 'I come, Lord Jesus!' Relapsing into quietude, I felt all but dismissed. It had the effect of so far arousing me, that I got power to speak, and called to my kindred, who came around me in surprise and anticipation. I took leave of them. I told one to be watchful, and spoke to the others, till power of speech again forsook me. As I lay I drew my hand to my breast to examine its beatings. I felt they were small and weak, and I was content, for I should soon be in another world. I was even anxious to die; for I feared lest, living again, I might lose what now seemed so sure. Then it was that a new order of feelings came over me. I had the most extraordinary sense of the bodily presence of the Power of Darkness standing by the side of my bed; not that I imagined that I saw anything, but I felt as if I could have put my hand on the very spot where he stood, and I shrank from that side with horror and loathing. But, blessed be God! on the opposite side stood, equally revealed to my spiritual senses, the Power unto Salvation, the very embodiment of love; and to this I turned as to a refuge. I shrank from the Evil One, and poured out my prayers to Christ, whose protection was evident to me. Thus I lay, when, all of a sudden, the most brilliant light darted into the room, and filled me with astonishment. Now, I thought, the time is surely at hand. God is visibly making manifest his approach. Quickly will the angels of God be descending, and I shall behold my Redeemer. By the vigour thus imparted I was enabled to sit up in bed, and with a feeling like that which Lazarus might have experienced, conscious of a supernatural Presence, I called out to my friends, 'Did you not see the light?' Next minute the impression came over me that I was yet to live; and at the same time, inspired with the certainty of knowing what I ought to take, I told my assistant to bring me forty drops of the tincture of opium, and twenty drops of the muriated tincture of iron, and to repeat the dose every twenty minutes. After taking the first dose, I continued sitting in bed; feeling as though entranced; and what is singular, my arms, when extended at an early part of the evening, had remained so, evincing the cataleptic state. I took the second dose, and lay down. These doses, so large that my assistant afterwards wondered what could have possessed him to give them, were the means of my recovery. After a miserable interval, during which the body seemed to be sinking into corruption, and the mind itself seemed to have lost all power of joy or sorrow, hope or fear, a profound sleep closed my eyes. It lasted upwards of twelve hours, and, awaking as from a dream, there remained no trace of my former state, except extreme debility. I never had the slightest relapse, but made rapid progress in recovery."

Dr. James Hamilton, the editor of this memoir, when commenting upon the preceding narrative, observes that there are

"One or two circumstances of which an ordinary spectator may possibly judge as accurately as the patient himself, with all his professional training.

"For instance, it was at the close of a laborious day, and when excessively fatigued, that Mr. Williams was first seized with those singular sensations in his head, and with the brilliant accompanying ideas. Now, to say nothing of any intermediate cause, such as determination of blood to the brain, we know that excessive application or exhaustion is not unfrequently followed by similar odd sensations. Dr. Moore mentions Dr. Isaac Watts, who, after great exertion of mind, thought his head too large to allow him to pass out at the study door; as also the case of a gentleman who, after delivering a lecture at the College of Surgeons, said that his head felt as if it filled the room.* With Mr. Williams the sensation was 'as though torrents of air were rushing into his brain, and the head itself expanding.' Nor do we

* The Power of the Soul over the Body. By George Moore, M.D. Fourth edition, p. 264.

suppose that it is at all uncommon for nervous exhaustion to be followed by such cataleptic seizures as Mr. Williams experienced, when his eyes were fixed, and when he had lost the power of speech, as well as voluntary respiration. The 'inspired certainty' with which he prescribed for himself the tonic opiate, need not surprise us. Suggested by some constitutional craving, invalids often fancy that if they could only obtain a given antidote, they would instantly be well. And they frequently are right. Sometimes the specific is a strange one, and would not readily have occurred to a man of science. In the present instance we presume that science would have countersigned the patient's prescription, had it only known all the circumstances; but then it must be remembered that in the present instance the patient himself was a doctor.

"'Intense mental conceptions so strongly impressed upon the mind as, for the moment, to be believed to have a real existence,' are amongst the most frequent spectral illusions.* As coming near this class, we must regard that 'extraordinary sense of the bodily presence of the Power of Darkness standing by the side of his bed,' which filled the imagination of the patient towards the close of his illness, as well as the brilliant light which followed. To bystanders no light was visible, no presence was palpable. Unlike the voice and the light on the road to Damascus, which the spectators heard and saw, these manifestations were confined to the individual's own mind.

"Still these ideas were substantially correct. Disease might embody them in forms too material; and yet they were truths. It was true that sins unnumbered stood chargeable against one who had hitherto lived without God in the world. It was true that God was offended, and death was coming. It was true that boundless dismay and terror environed the Christless transgressor. The name of Jesus had no more effect in tranquillizing the conscience and kindling hope than that blessed name should ever have. And the instinct which shrank from the Power of Darkness and cried to Jesus for protection, was itself a token that a new life was dawning. There might be nervous excitement, but there was also a spiritual awakening. There might be morbid sensations; but the pervading conviction was scriptural, and the consequent change of thought and feeling was permanent. That change we shall leave Mr. Williams to describe.

"'It was on the fifteenth day of September 1846 that I was taken ill. It is now September 1847 when I am writing this. The delightful feelings of the first few days of convalescence I remember well. Joyfully exulting in the interposition of Divine Providence and mercy, which had brought me out of thick darkness into the glorious light of truth, O what a heaven flitted through my soul! Holiness with its celestial gilding seemed to tinge every object around me. The world was no longer the same world; its people no longer the same beings. Myself and my fellows I no longer regarded as creatures of a moment's duration, but I saw eternity impressed as a seal on the whole generation

* See Hibbert on Apparitions. Abercrombie on the Intellectual Powers, Part 3.

of men. The universe was no longer a confused assemblage of indistinct parts, moving towards a gloomy terminus, but, as far as the Divine purposes were concerned, a bright whole of uniform perfection, and the entire expanse filled with love, unbounded love. God himself seemed to move everywhere. All was joy to my soul. I looked on myself as a brand plucked from the burning, and rejoiced in the sure hope of salvation. Jesus was most precious to me,—my glory and infinite joy. The Bible, hitherto a sealed book, was now a river to my thirsty soul. I was astounded at its contents. As I turned over its pages, wonder upon wonder ravished my delighted heart. I felt that I would care to live only for the sake of reading it. It was a glorious light. At times its heavenly rays would subdue me into a mellow and peaceful benignity; at others, rouse me into ecstatic bliss. Everywhere was the authority, the love, of God recognised. Its power to command my obedience was as the thunder-clad arm of Omnipotence; and its pleadings for holiness were as the gentle whisperings of love, to which my heart, my mind, my soul answered assent. How I wondered at my former darkness! How amazed did I feel that the precious light had so long shone in my way, and I never had perceived it! I resolved to make it the absolute rule of my life.

"'These first days were as though they had been a foretaste of heavenly peace. Never shall I forget my first mortification at finding that sin still existed within me. There had been no actual committal of an offence that my conscience charged me with; yet a sudden and unexpected change had come over me. There was a cloudiness in my mind; my faith was dim; my heart had ceased to exult. It was as though all had been a bright and glorious dream, and I had now awakened to the stern realities of a cold and miserable world. Alas, the bitterness of that moment! I strove to recall my hopes—they seemed delusion. I read my Bible—the bright revealing light which had heretofore almost made the very print more clear was gone; and, although I still knew it to be the Word of God, the page had ceased to enkindle rapture or inspire emotion. I knew not how to account for this state. I had believed that the work of change and renovation had been completed, at least carried to so high a degree that it was impossible I could wilfully sin against God again. I abhorred the thought, yet here I was in darkness, and sin palpably abounding in my heart. How sad was the sight of myself! It was the first glimpse at the inherent corruption and original depravity of my heart. It was the first of a series of painful but important lessons which convinced me that God had only hitherto instructed me in the first principles, and laid the foundation for my faith; but that the work of grace had to be carried on, and an absolute change of heart effected, by many a severe and fiery ordeal.

"'In the course of weeks, I was enabled to take a trip into North Wales; here my connexion with the world was first re-established. All the avocations of man, that were apart from his religious. duties, appeared to me to have vanity legibly stamped on them. On my route I stopped a short time in Liverpool, but the bustle and commotion excited no pleasurable sympathy, for I felt that it was all vanity. The whirl, the din, the confusion, all told me of the world's spirit, and in the countenance of the busy throng I could not read one expression in unison with my own feelings, or which came home to my heart. At Beaumaris I abode at a commercial hotel, and there, in the presence of the usual visitants of an inn, I took out my Bible, glorying in the thought that I was thus unfurling Christ's banner. One of the company entered into conversation, and boasted of his religious acquisitions, and of the high position he held in the church to which he belonged as teacher and deacon. But gradually he drank to inebriation. I was glad to find a room to myself, and in dejection to ponder over this first instance of a false professor.

"'My stay in North Wales, especially my visit to Llanberis and Snowdon, afforded my mind the healthful occupation of contemplating and adoring God as revealed in his works. To me the God of nature and the God of revelation now were one, and I began more sensibly to feel the relation wherein we stand to God by the conjoint link of creation and redemption. How glorious to know that a pathway had been opened for the rebellious sinner to the favour of the great Eternal, whose hand had formed the mighty fabric of the universe, and who had given the being and life we enjoy, but from whom I had so long been severed, and to whom I had never felt my relation, nor acknowledged my obedience! But the great Eternal was now the Lord my God, and I, the creature of his hand, could, through the Redeemer, look up and believe that the Power which guided the planets in their course would direct me in all my ways, and preserve me by his providential care. I felt that he had first loved me. I felt that God so loved the world as to give his only begotten Son, that whosoever believeth in him should not perish, but have everlasting life. I felt that it is the First and the Last who there expresses his care for all the family of man, including myself, a worm so insignificant. At that mercy I could only wonder and adore, and, with faint conceptions of his love and grace, I could but humble myself before him.'"

We place upon record the preceding extracts from the two volumes referred to, as matter for future psychological analysis and comment. They cannot prove otherwise than of deep interest to all engaged in the study of medical-psychology.

